# 

**Published:** 1885-10

**Authors:** 


### CONTENTS.

Original Articles.—Medical Etiquette—The Proper Mission of the Obstetric Forceps—A Run of Bad Cases—Malarial Hæmaturia—Emmet's Operation followed by Pregnancy in Three Months ......... 159–174

Cullings from Contemporaries.—Prolonged Anæsthesia from Cocaine—Operative Treatment of Acute Intestinal Obstruction—Abortion of Typhoid Fever—Two New Local Anæsthetics................ 174–179

Correspondence.—Treatment of Eczema—Dr. McLaughlin's Reply to Dr. Swearingen's Criticism .. 180–182

Society Notes.—Errors in Transactions T. S. M. A.—Physicians' Mutual Benefit Association ...... 186–187

Bibliography.—Transactions T. S. M. A., 1885 ...... 188

Editorial—Dengue Epidemic in Texas—Disseminating Truth and doing good *a la mode*—The Ninth International Congress—Professional Sentiment as Evidenced by the Medical Press—A Disgusted Philanthropist ................................ 190–199

Melange—A New Speculum—Another Good Man Gone—Witty—A Benefit—Hymenial, etc ....... 202

Advertisers' Notices ............................ 202

